# A History of a Rupture of the Uterus during Labour

**Published:** 1814-09

**Authors:** John Ramsbotham


*For the Medical and Physical Journal.*

### *A History of a Rupture of the Uterus during Labour*; by JOHN RAMSBOTHAM, M.D.

MRS. D. æt. 43, fell into labour of her sixth child early on the morning of Sunday the 10th of July. Her preceding labours had been natural but lingering, and her children had been born alive, except the first: in that instance the labour was of longer continuance, and she suffered more from its effects than afterwards; yet it was at length completed by the natural powers. Her general health, naturally somewhat delicate, had been as good as usual during her pregnancy; and, upon the whole, she had passed through that state with as little general inconvenience as on former occasions. Previous to her falling into actual labour, however, she had several times been attacked with slight recurrent pains in the night, which had induced her to apprise her professional attendant of her situation; and partly from this circumstance, to which in her former pregnancies she had been unaccustomed, and partly from the suspicion that she might have twins, she became low-spirited, and had got her mind impressed with the idea that she should not do well.

About seven o'clock on the morning above-mentioned, the immediate attendance of her accoucheur was requested. On his arrival, he was informed that the labour-pains had commenced a few hours before, and had been gradually increasing in strength, so that they appeared to be regularly established; on an examination, the membranes were found considerably protruded through the os uteri, but the head of the child was above the brim of the pelvis, and could scarcely be felt. After a little time the membranes gave way, the waters were suddenly discharged in considerable quantity, and the head was observed to be descending, yet still remaining high; it was however presently found to advance by the assistance of the pains, and, under the present circumstances, the patient was promised as favourable a delivery as in her preceding labours. For some hours the labour appeared to go on in a regular natural course, the head of the child advanced in the pelvis, yet slowly, and the patient kept up her spirits: but in the early part of the afternoon, she complained of an unusual sort of pain, shooting from the stomach to the back (as she expressed herself) which she described as a sort of *stitch*, very different from any pain she had experienced in her former labours; this new pain was accompanied with some difficulty of breathing, occasional hiccough, slight vomitings of a glary mucous fluid,

 and and repeated eructations, which were extremely troublesome to her, and she complained much afterward of the inconvenience produced by them. The regular labour-pains continued to be sufficiently strong, and of the expulsive kind, and did not appear, *at present*, to be at all affected by the accidental occurrence of these new and unusual symptoms; they were therefore attributed to the effects of the process of labour upon a delicate frame and to wind; but though the pains did continue strong and expulsive, they did not produce much advance of the head, which seemed wedged in the pelvis. Besides, this general state of the patient occasioned the less uneasiness, as her former labours had uniformly been tedious, and had called forth great exertions before they were completed. Towards evening, still complaining of being much inconvenienced by this unusual pain or stitch, six or eight ounces of blood were taken from the arm; during this operation, the patient was set upright in an easy chair, and remained in that posture for some time. Hitherto the labour-pains had been strong and frequent, yet had of late produced little effect upon the head in its advance through the pelvis; but some time hence, they were observed to diminish in strength, and to have longer intervals, still, however, retaining the characters of expulsive pains; the patient had also occasionally mentioned a sense of faintness, which was supposed to arise from the loss of blood from the arm, for as yet there had been only a trifling-coloured discharge from the vagina. The extremities were cold, and the pulse had become languid. The accoucheur had occasionally applied his hand upon the abdomen, and had noticed an uncommon irregularity of the abdominal tumour, which assumed a character very different from what he had ever observed in natural labours: the patient herself was also satisfied there was something peculiar in her situation, and had repeatedly enquired whether such peculiar sensations might not be occasioned by twins. She still suffered considerably from frequent eructations, and continued to ascribe a great part of her sufferings to wind, with which she had hitherto been much troubled.

As the labour seemed to have made no progress towards a conclusion for some hours past, and as the patient was considered, from the general symptoms, to be in a state of danger, a consultation was requested, and, between nine and ten in the evening, the husband of the lady waited on me, and urgently desired my immediate attendance. On my arrival at the bed-side of my patient, I found her under a state of considerable exhaustion, evidently unconnected with hæmorrhage or other external symptom, and apparently

 not not produced by the common effects and course of a lingering protracted labour; her countenance was very much dejected; her spirits depressed; her breathing frequent and laboured; her pulse small and rapid; and her hands cold and clammy. She had occasional slight labour-pains, but they were not at this time of the expulsive kind, and complained much of a sense of *sinking*, which is very inadequately expressed by the term faintness; upon the whole, the quantity of coloured discharge from the vagina had been, as yet, inconsiderable. On examining the abdomen, its general enlargement appeared as great as under the common circumstances of labour, but I could detect no uniform uterine tumour; and, on pressure with the hand, various inequalities in its general extension were distinctly observable. The head of the child was low down in the pelvis, but it had not effected its turn, so as to place the occiput under the pubis.

Under such unfavourable appearances, I recommended immediate delivery; and by the assistance of the vectis, the head was extracted within a very moderate space of time; some expulsive efforts soon followed, which essentially forwarded the delivery of the shoulders, body, and feet, of a still-born infant. Immediately after the birth of the child, a considerable discharge of fluid blood took place, which, if continued, under the exhausted state of the patient, seemed quite inconsistent with any chance of recovery. This occurrence induced me to attempt, without loss of time, the removal of the placenta. On examining the vagina, all the parts felt particularly flaccid, and on carrying my hand upward, guided by the funis, I was soon satisfied that it was not within the uterus, but must have passed into the general cavity of the abdomen. On bringing the funis gently to its bearing, I observed the placenta to descend gradually; and, receiving it in my hand, I readily withdrew it. During this part of the operation, I could perceive the contracted uterus before my hand on the fore and lower part of the abdomen. I afterwards with my hand examined the external surface of the abdomen, and found the uterus as well contracted as under the common circumstances of labour, and in its usual situation just above the pubis. After delivery, the patient remained in a languid exhausted state for some time, but after the exhibition of some cordials and an opiate occasionally, she considerably revived, spoke with cheerfulness, and expressed herself to be quite relieved; warmth also returned into her extremities. During the remainder of the night she was free from pain, and towards morning got a little unrefreshing sleep. On visiting her the next morning,

I found

I found her cheerful, and free from pain, except on pressing the belly; the countenance had resumed its natural appearance; she had passed her urine without any difficulty; but her pulse was small and quick. An opening mixture was directed in divided doses to relieve the bowels. At nine in the evening, she continued very much in the same state; but the pulse was observed to be increasing in velocity, and the belly to have become more tense and tender. The bowels had not as yet been relieved; clysters were therefore desired to be occasionally injected. After the middle of the night, she changed for the worse; the stomach began to reject whatever was taken into it; the restlessness and anxiety increased; the belly became more tense and tender; towards morning the powers of life were observed gradually to decline, and she died about eleven on the Tuesday forenoon.

Leave was obtained to inspect the body the day following. On dividing the abdominal parietes, the uterus was observed to be well contracted, and of its usual shape; appearances of inflammatory affection shewed themselves on the peritoneal coat of the intestinal canal, and within its folds were small coagula of blood; on raising the uterus forward, a fissure or rent was found on its back part near the cervix, sufficiently large to admit the hand, running nearly from side to side, but which had not implicated the os uteri, vagina, or rectum, in the ravages it had made. The projection of the sacrum was also greater than in a well-formed pelvis.

Through this rent in the back part of the cervix uteri, the contractions of its fundus and body had propelled all the child with the placenta into the cavity of the abdomen except its head, which was fast jammed in the pelvis and could not retreat. The rupture most probably took place in the early part of the afternoon of Sunday; when the patient complained of that peculiar pain or stitch, followed by a sense of sinking, and other unpleasant symptoms; and the expulsive pains which continued after this event, without making any impression on the head in its advance through the pelvis, were those uterine contractions by which the child was expelled its cavity, and escaped through the rent into the belly. The irregularities before noticed in the abdominal extension, were the limbs of the child felt through the abdominal muscles. By referring to the pages of your valuable Journal for October 1813, your readers will find another account of a ruptured uterus, with some remarks; forming, with the present case, a tolerable history of this unfortunate occurrence.

JOHN RAMSBOTHAM.

